# Supplementary material for: Influence of Sporulation Temperature on Germination and Growth of B. weihenstephanensis Strains in Specific Nutrients and in an Extended Shelf-Life Refrigerated Matrix Under Commercial Pasteurization and Storage Conditions
Source: Foods. 2024 Oct 28;13(21):3434. doi: 10.3390/foods13213434 (PMC11545089; doi:10.3390/foods13213434)
Supplement: Supplementary file 1 [file foods-13-03434-s001.zip › foods-3251129-supplementary.pdf]

**Figure S1.** Germination curves of non-heat-activated spores of strains WSBC 10202, WSBC 10204, and SC produced at $T_{min}$ (blue), $T_{opt}$ (green), and $T_{max}$ (red) in the indicated nutrients at 25 °C. Germination represents the percentage of $OD_{600}$ decrease ($OD_t/OD_0 \times 100$) over time. Data in the figures correspond to the mean and standard deviation calculated from three biological replicates.

**Figure S2.** Germination curves of heat-activated (80 ºC, 10 min) spores of strains WSBC 10202, WSBC 10204, and SC produced at $T_{min}$ (blue), $T_{opt}$ (green), and $T_{max}$ (red) in the indicated nutrients at 25 °C. Germination represents the percentage of $OD_{600}$ decrease ($OD_t/OD_0 \times 100$) over time. Data in the figures correspond to the mean and standard deviation calculated from three biological replicates.

**Figure S3.** Germination curves of untreated (row A) and heat-treated (row B: 80 ºC, 10 min; row C: 90 ºC, 10 min) spores of strains WSBC 10202, WSBC 10204, and SC produced at $T_{min}$ (blue), $T_{opt}$ (green), and $T_{max}$ (red) in an ESL matrix at 25 °C. Germination represents the percentage of $OD_{600}$ fall ($OD_t/OD_0 \times 100$) over time. Data in the figures correspond to averages and standard deviations calculated from three biological replicates.

**Table S1.** $R^2$ and RMSE obtained from fitting germination curves of non-activated and heat-activated (80 ºC, 10 min) spores of strains WSBC 10202, WSBC 10204, and SC produced at different temperatures in the indicated nutrients at 25 ºC (Figure S1, Figure S2) to the One-Phase Decay model (Eq. 1).

**Figure S1.**

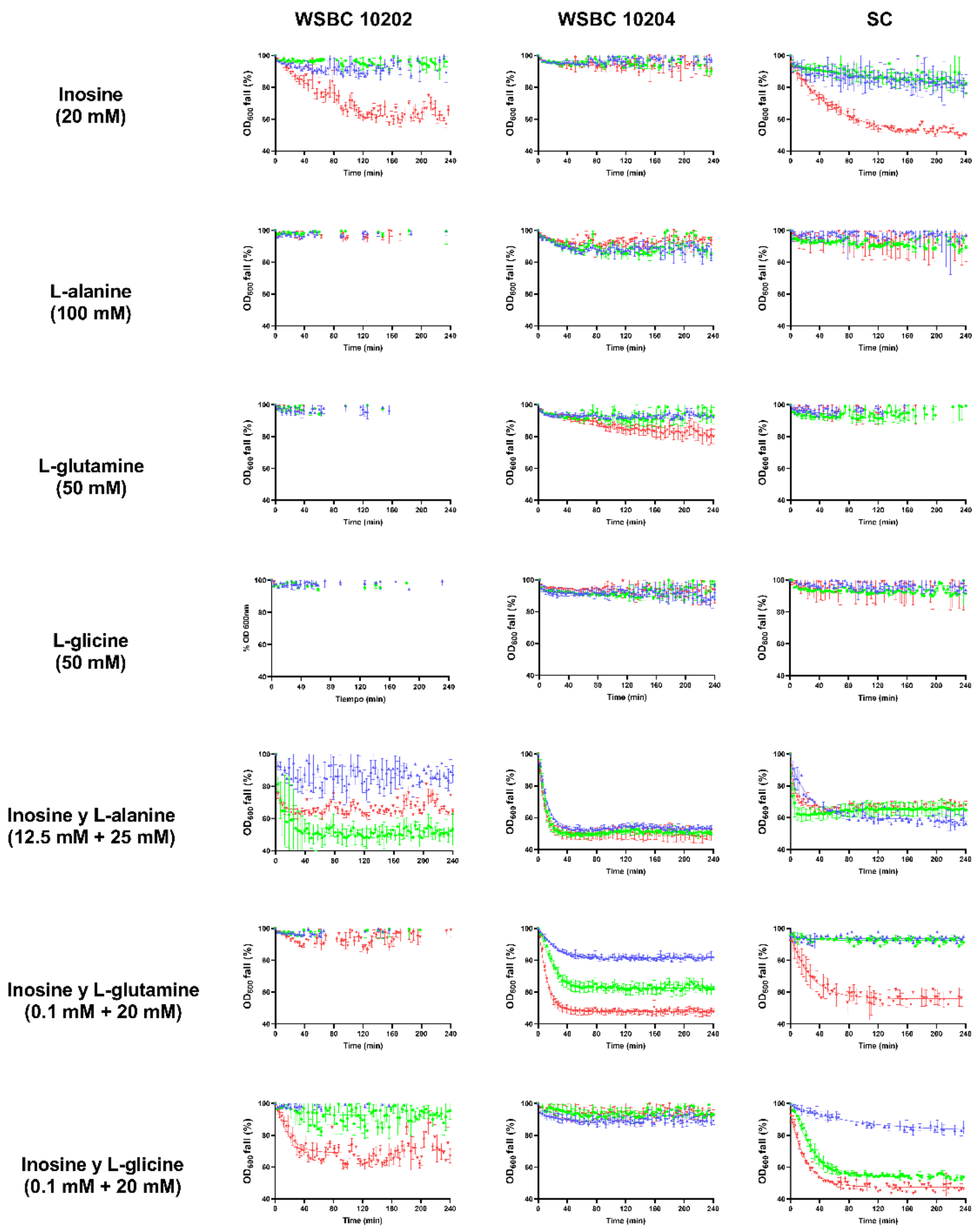
WSBC 10202
WSBC 10204
SC
Inosine
(20 mM)
L-alanine
(100 mM)
L-glutamine
(50 mM)
L-glicine
(50 mM)
Inosine y L-alanine
(12.5 mM + 25 mM)
Inosine y L-glutamine
(0.1 mM + 20 mM)
Inosine y L-glicine
(0.1 mM + 20 mM)
OD600 fall (%)
% OD 600nm
Time (min)
Tiempo (min)

**Figure S2.**

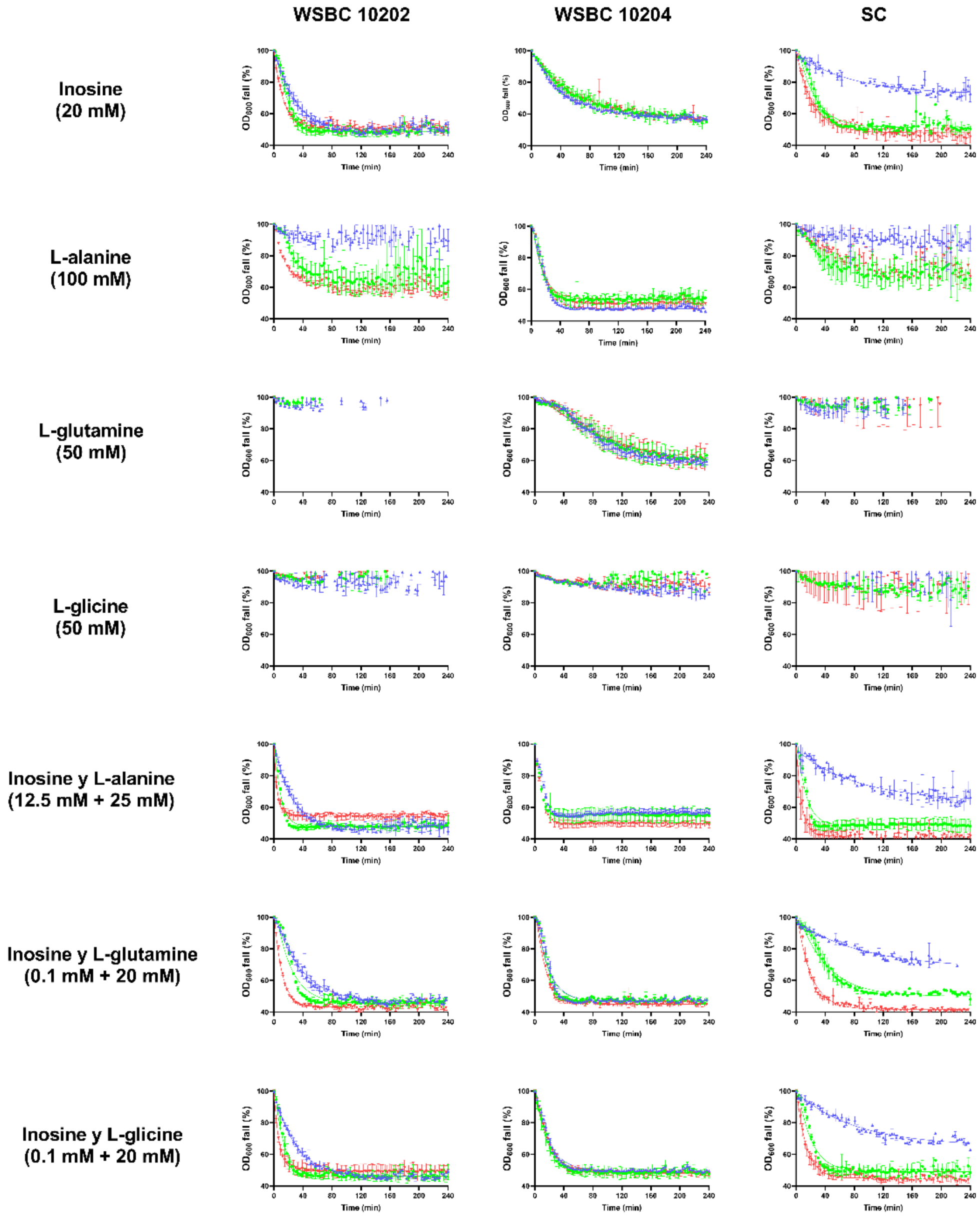
WSBC 10202
WSBC 10204
SC
Inosine
(20 mM)
L-alanine
(100 mM)
L-glutamine
(50 mM)
L-glicine
(50 mM)
Inosine y L-alanine
(12.5 mM + 25 mM)
Inosine y L-glutamine
(0.1 mM + 20 mM)
Inosine y L-glicine
(0.1 mM + 20 mM)
OD600 fall (%)
Time (min)

**Figure S3**

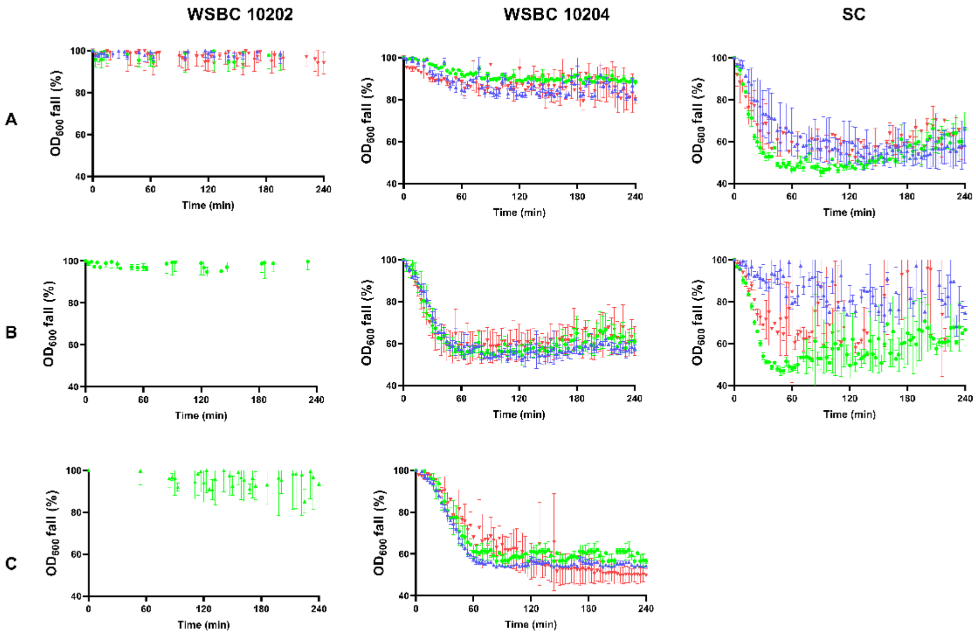
WSBC 10202
WSBC 10204
SC
A
B
C
OD600 fall (%)
Time (min)
0
60
120
180
240
40
60
80
100

**Table S1.**

| | | WSBC 10202 | | | | WSBC 10204 | | | | SC | | | |
|---|---|---|---|---|---|---|---|---|---|---|---|---|---|
| | | Non-activated | | Heat-activated | | Non-activated | | Heat-activated | | Non-activated | | Heat-activated | |
| **Germinant** | **Sporulation temperature** | **$R^2$** | **RMSE** | **$R^2$** | **RMSE** | **$R^2$** | **RMSE** | **$R^2$** | **RMSE** | **$R^2$** | **RMSE** | **$R^2$** | **RMSE** |
| **Inosine (20 mM)** | **$T_{min}$** | N.M. | N.M. | 0.956 | 2.435 | N.M. | N.M. | 0.991 | 0.991 | 0.859 | 1.885 | 0.899 | 2.125 |
| | **$T_{opt}$** | N.M. | N.M. | 0.948 | 2.690 | N.M. | N.M. | 0.983 | 1.487 | 0.949 | 1.132 | 0.961 | 2.932 |
| | **$T_{max}$** | 0.965 | 2.233 | 0.954 | 1.883 | N.M. | N.M. | 0.988 | 1.236 | 0.974 | 1.473 | 0.961 | 2.362 |
| **L-alanine (100 mM)** | **$T_{min}$** | N.M. | N.M. | 0.968 | 1.551 | N.M. | N.M. | 0.966 | 2.097 | N.M. | N.M. | N.M. | N.M. |
| | **$T_{opt}$** | N.M. | N.M. | 0.964 | 2.298 | N.M. | N.M. | 0.949 | 2.215 | N.M. | N.M. | 0.970 | 1.888 |
| | **$T_{max}$** | N.M. | N.M. | 0.972 | 1.836 | N.M. | N.M. | 0.979 | 1.546 | N.M. | N.M. | 0.954 | 1.985 |
| **L-glutamine (50 mM)** | **$T_{min}$** | N.M. | N.M. | N.M. | N.M. | N.M. | N.M. | 0.969 | 2.450 | N.M. | N.M. | N.M. | N.M. |
| | **$T_{opt}$** | N.M. | N.M. | N.M. | N.M. | N.M. | N.M. | 0.960 | 2.531 | N.M. | N.M. | N.M. | N.M. |
| | **$T_{max}$** | N.M. | N.M. | N.M. | N.M. | N.M. | N.M. | 0.973 | 1.996 | N.M. | N.M. | N.M. | N.M. |
| **L-glycine (50 mM)** | **$T_{min}$** | N.M. | N.M. | N.M. | N.M. | N.M. | N.M. | N.M. | N.M. | N.M. | N.M. | N.M. | N.M. |
| | **$T_{opt}$** | N.M. | N.M. | N.M. | N.M. | N.M. | N.M. | N.M. | N.M. | N.M. | N.M. | N.M. | N.M. |
| | **$T_{max}$** | N.M. | N.M. | N.M. | N.M. | N.M. | N.M. | N.M. | N.M. | N.M. | N.M. | N.M. | N.M. |
| **Inosine (12.5 mM) + L-alanine (25 mM)** | **$T_{min}$** | 0.944 | 1.570 | 0.970 | 2.018 | 0.970 | 1.412 | 0.953 | 1.642 | 0.928 | 2.553 | 0.923 | 2.021 |
| | **$T_{opt}$** | 0.986 | 1.105 | 0.952 | 1.961 | 0.965 | 1.555 | 0.978 | 1.041 | 0.986 | 0.664 | 0.941 | 2.499 |
| | **$T_{max}$** | 0.979 | 0.810 | 0.969 | 1.056 | 0.958 | 1.578 | 0.990 | 0.771 | 0.933 | 1.941 | 0.939 | 2.183 |
| **Inosine (0.1 mM) + L-glutamine (20 mM)** | **$T_{min}$** | N.M. | N.M. | 0.981 | 2.010 | 0.928 | 1.084 | 0.955 | 2.590 | N.M. | N.M. | 0.947 | 1.900 |
| | **$T_{opt}$** | N.M. | N.M. | 0.939 | 3.552 | 0.955 | 1.788 | 0.946 | 2.864 | N.M. | N.M. | 0.966 | 2.842 |
| | **$T_{max}$** | N.M. | N.M. | 0.975 | 1.549 | 0.980 | 1.359 | 0.965 | 2.186 | 0.963 | 1.911 | 0.979 | 2.158 |
| **Inosine (0.1 mM) + L-glycine (20 mM)** | **$T_{min}$** | N.M. | N.M. | 0.988 | 1.492 | N.M. | N.M. | 0.981 | 1.482 | 0.928 | 2.553 | 0.965 | 1.813 |
| | **$T_{opt}$** | N.M. | N.M. | 0.966 | 2.122 | N.M. | N.M. | 0.982 | 1.377 | 0.986 | 0.664 | 0.942 | 3.228 |
| | **$T_{max}$** | 0.984 | 1.173 | 0.986 | 0.929 | N.M. | N.M. | 0.990 | 1.048 | 0.933 | 1.941 | 0.966 | 1.982 |

N.M.: not modelled. OD600 values did not decrease more than 15%.
